# CD4^+^ T-cell survival in the GI tract requires dectin-1 during fungal infection

**DOI:** 10.1038/mi.2015.79

**Published:** 2015-09-09

**Authors:** R A Drummond, I M Dambuza, S Vautier, J A Taylor, D M Reid, C C Bain, D M Underhill, D Masopust, D H Kaplan, G D Brown

**Affiliations:** 1 Aberdeen Fungal Group, Institute of Medical Sciences, University of Aberdeen, Aberdeen, UK; 2 Institute of Infection, Immunity and Inflammation, College of Veterinary, Medical and Life Science, University of Glasgow, Scotland, UK; 3 Division of Immunology, The Inflammatory Bowel and Immunobiology Research Institute, Cedars-Sinai Medical Centre, Los Angeles, California, USA; 4 Department of Microbiology, Centre for Immunology, University of Minnesota Medical School, Minneapolis, Minnesota, USA; 5 Department of Dermatology, Centre for Immunology, University of Minnesota, Minneapolis, Minnesota, USA

## Abstract

Dectin-1 is an innate antifungal C-type lectin receptor necessary for protective antifungal immunity. We recently discovered that Dectin-1 is involved in controlling fungal infections of the gastrointestinal (GI) tract, but how this C-type lectin receptor mediates these activities is unknown. Here, we show that Dectin-1 is essential for driving fungal-specific CD4^+^ T-cell responses in the GI tract. Loss of Dectin-1 resulted in abrogated dendritic cell responses in the mesenteric lymph nodes (mLNs) and defective T-cell co-stimulation, causing substantial increases in CD4^+^ T-cell apoptosis and reductions in the cellularity of GI-associated lymphoid tissues. CD8^+^ T-cell responses were unaffected by Dectin-1 deficiency. These functions of Dectin-1 have significant implications for our understanding of intestinal immunity and susceptibility to fungal infections.

## Introduction

Innate recognition of fungi is primarily mediated by a large class of myeloid-expressed pattern recognition receptors, termed the C-type lectin receptors.^1^ Dectin-1 (gene symbol *Clec7a*) is the prototypical member of this family and is an essential component of the protective immune response to numerous fungal pathogens including *Candida albicans*,^2^ *Pneumocystis carinii,*^3^ and *Aspergillus fumigatus*.^4^ Dectin-1 binds exposed β-glucans in fungal cell walls and initiates complex intracellular signaling pathways through Syk kinase and CARD9 leading to phagocytosis of fungi, the respiratory burst, and pro-inflammatory cytokine production.^1^ Dectin-1 has also been shown to influence the development of adaptive antifungal immunity by driving Th17 polarization.^5^ More recently, C-type lectin receptors such as Dectin-2 (gene symbol *Clec4n*) have been implicated in anti-*Candida* immunity, in part through collaborative interactions with Dectin-1 and other pattern recognition receptors.^6, 7^

Fungi are now recognized as important components of the murine and human gastrointestinal (GI) microbiomes.^8, 9, 10^ In humans, *C. albicans* is a common GI commensal and this population is thought to serve as reservoir for systemic infections following invasion of GI tissues under conditions of immunosuppression.^11, 12, 13^ In addition to protecting against systemic fungal disease, we have shown that Dectin-1 has a crucial role in protecting the GI tract during systemic candidiasis and in inflammatory bowel disorders, although the Dectin-1-dependent mechanisms operating in this tissue are not understood.^8, 14^ In murine models of colitis, where DSS is used to disrupt the mucosal barrier, we found that Dectin-1^−/−^ mice exhibited enhanced inflammation if exposed to *Candida* during the induction of colitis. In line with the murine studies, we identified a single-nucleotide polymorphism in human *CLEC7A* (rs2078178) that significantly associated with ulcerative colitis.^8^ Moreover, we have found that Dectin-1^−/−^ animals systemically infected with *C. albicans* have increased GI fungal burdens and dysregulated cytokine production.^14^ These data therefore suggest that Dectin-1 has a crucial role in protecting the GI tract from fungal-mediated inflammation.

Our understanding of Dectin-1-dependent antifungal immunity has concentrated largely on its innate functions and we still know little about the influence of this receptor on adaptive immunity during infection. The role of Dectin-1 and other C-type lectin receptors in driving these responses is likely to be important for GI tract homeostasis, as defects in T-cell function within the human and mouse gut, particularly Th17 polarization, is associated with inflammatory bowel disease.^15^ We therefore examined the role of Dectin-1 in controlling T-cell activation in the GI tract during fungal infection.

## Results

### Dectin-1 controls GI antigen-specific CD4^+^ T-cell responses

**Figure 1:**
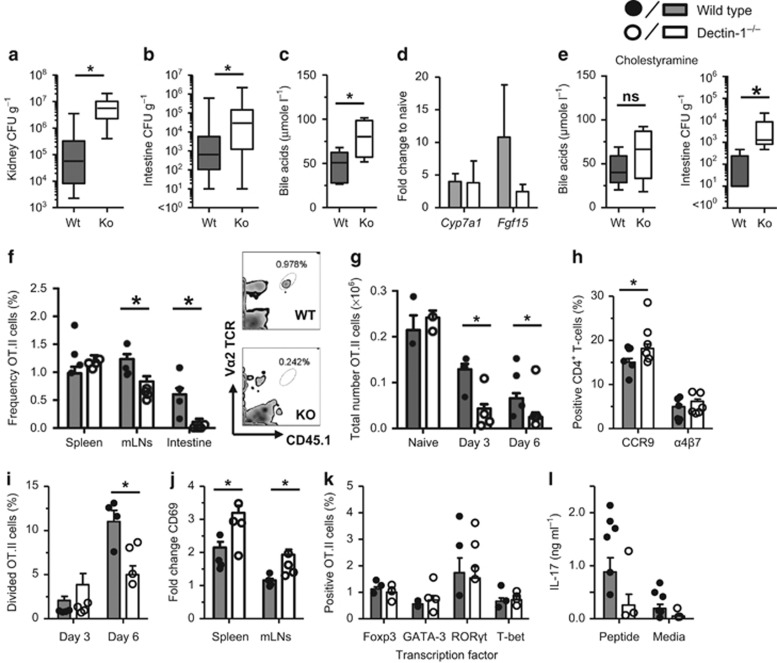
Dectin-1 is required for fungal-specific CD4^+^ T-cell responses in the GI tract. WT (filled bars/circles) and Dectin-1^−/−^ (clear bars/circles) animals were systemically infected via the tail vein with 2 × 10^5^ CFU *C. albicans* and analyzed at 72 h post infection for (**a**) kidney (WT *n*=25, KO *n*=25) and (**b**) small intestine fungal burdens (WT *n*=17, KO *n*=21). (**c**) Bile-acid concentration in the small intestine of infected WT (*n*=5) and Dectin-1 KO (*n*=6) mice. (**d**) The fold change (to naïve controls) in genes regulating bile-acid synthesis (*Cyp7a1*) or in bile-acid-negative feedback (*Fgf15*) were measured in 3 day infected livers and intestines (respectively) by quantitative PCR (*n*=3). (**e**) WT (*n*=10) and Dectin-1 KO (*n*=9) animals maintained on cholestyramine diets starting at 24 h pre-infection until 72 h post infection, when bile acids were measured as in (**c**), and intestine fungal burdens determined. In other experiments, 3 × 10^6^ OT.II T-cells were transferred into WT and Dectin-1^−/−^ animals, and then infected via the tail vein with 2 × 10^5^ CFU Calb-Ag and analyzed at 72 h post infection. (**f**) Frequency of OT.II T-cells (CD4^+^Vα2^+^CD45.1^+^) was assessed by flow cytometry in the spleen (*n*=12), mLNs (*n*=12), and small intestine (*n*=8). Example plots are shown for the small intestine and are gated on CD4^+^ lymphocytes. (**g**) Total number of OT.II T-cells in the mLN at the indicated time points post infection. (**h**) Expression of CCR9 (WT *n*=20, KO *n*=18) and α4β7 (WT *n*=16, KO *n*=14) by CD4^+^ T-cells in the mLN. (**i**) The dilution of carboxyfluorescein succinimidyl ester (CFSE) was used to measure proliferation of OT.II cells at day 3 (*n*=12) and day 6 (WT *n*=12, KO *n*=10) post infection in the mLNs. Graph shows percentage of cells that diluted CFSE; see Supplementary Figure S2 for data expressed as the division index.^36^ (**j**) Activation of OT.II cells in the spleen (*n*=8) and mLN (*n*=12) was determined by staining for CD69 and comparing with naive controls. (**k**) Expression of indicated transcription factors by OT.II cells in the mLN was analyzed by intracellular staining and flow cytometry at day 3 post infection (WT *n*=6, KO *n*=8). (**l**) 2 × 10^6^ cells from mLN suspensions at day 3 post infection were restimulated *in vitro* with OVA_323-339_ peptide and IL-17 in the supernatant measured by ELISA (WT *n*=10, KO *n*=6). Bar charts show pooled data (2–4 experiments); overlaid dot plots show a single representative experiment. TCR, T-cell receptor. *P<0.05.

We systemically infected wild-type (WT) and *Clec7a*^−/−^ (Dectin-1 KO) animals with *C. albicans* and found significant increases in fungal burdens in the kidneys and small intestine of Dectin-1^−/−^ mice (Figure 1a, b), as we had observed previously.^2, 14^ In addition to enhanced infection of the small intestine, infected Dectin-1 KO mice had significantly higher bile-acid levels in the small intestine (Figure 1c).^14^ Despite an increase in the bile-acid-synthesizing enzyme *Cyp7a1* (ref. 16) in the liver during infection, Dectin-1 KO mice displayed poor upregulation of the intestinal negative feedback regulator, *Fgf15* (ref. 17) (Figure 1d), suggesting that the increased bile acids were the result of a defect in this negative feedback loop. However, including the bile-acid-sequesterant cholestyramine^18^ in the diet lowered bile acids to WT levels but did not significantly alter fungal burdens in the small intestine (Figure 1e). This suggests that the altered bile-acid concentration within the Dectin-1 KO mice is not responsible for the lack of control of fungal infection in these tissues.

To characterize the role of Dectin-1 in antigen-specific T-cell responses during infection, we made use of the OT.I/OT.II T-cell transgenic system and a previously characterized strain of *C. albicans* expressing ovalbumin (OVA) peptides (Calb-Ag),^19^ as there are no T-cell receptor transgenic models specific for *C. albicans* currently available. We adoptively transferred CD4^+^ OT.II cells into WT and Dectin-1^−/−^ animals and then 24 h later infected these mice with Calb-Ag. Responding OT.II cells were subsequently analyzed in various tissues by flow cytometry. We found a specific reduction in the frequency and number of OT.II T-cells within the intestinal draining mesenteric lymph nodes (mLN) and small intestine of Dectin-1^−/−^ mice compared with WT animals (Figure 1f, g), despite increased expression of gut-homing receptors CCR9 and α4β7 by CD4^+^ T-cells in the mLN (Figure 1h). In contrast, we found no reduction in the frequency of these cells in the spleen (Figure 1f), kidney-draining (renal) lymph nodes during infection, or in these tissues in naive animals (Supplementary Figure 1a, b online).

Owing to the lack of OT.II T-cells in the infected Dectin-1 KO small intestine, we were unable to analyse any functional parameters in this tissue and therefore concentrated our analysis on the mLNs. OT.II responses were generally weak in this LN when compared with the renal LNs and spleen at this time point, which are likely to be related to the lower fungal burdens found in the intestine (Figure 1b). Nevertheless, we found that OT.II responses in the mLN were differentially regulated in the Dectin-1 KO mice. Monitoring cell division by carboxyfluorescein succinimidyl ester dilution, OT.II proliferation increased from day 3 to day 6 in the WT mLNs. In contrast, the Dectin-1 KO mice did not show any increase in proliferation over this time period (Figure 1i and Supplementary Figure S2). Similar to the defect in frequency, this reduction in proliferation of antigen-specific CD4^+^ T-cells was only evident in the GI tract, as no defects were detected in the spleen or renal lymph nodes of Dectin-1 KO mice (Supplementary Figure S1c, d). Interestingly, Dectin-1 deficiency did not reduce OT.II cell activation, measured by CD69 upregulation, which was enhanced over WT in both the mLN and the spleen (Figure 1j). Moreover, Dectin-1 was not required for T-cell polarization, measured by master transcription factor expression, in any of the sites analyzed (Figure 1k and Supplementary Figure S1c, d). However, we did detect a non-significant reduction in interleukin (IL)-17 production from mLN cells following *in vitro* restimulation (Figure 1l), consistent with previous reports showing a role for this receptor in driving Th17 responses.^5^

### Dectin-1 is not required for antigen-specific CD8^+^ T-cell responses in the GI tract

**Figure 2:**
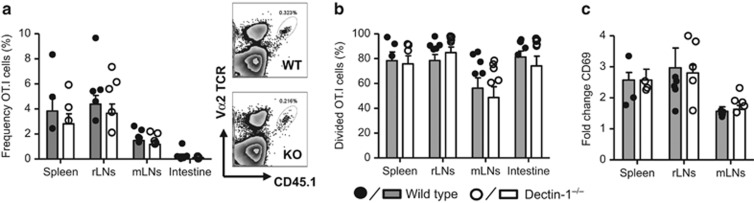
Dectin-1 is not required for antigen-specific CD8^+^ T-cell responses. (**a**) Frequency, (**b**) division, and (**c**) activation of OT.I T-cells (CD8^+^Vα2^+^CD45.1^+^) was assessed as in Figure 1 in the indicated tissues (WT *n*=10, KO *n*=9) at day 3 post infection. Bar charts show pooled data (two experiments); overlaid dot plots show a single representative experiment. TCR, T-cell receptor.

To explore the effect of Dectin-1 deficiency on CD8^+^ T-cell responses during fungal infection, we adoptively transferred CD8^+^ OT.I cells into WT and Dectin-1^−/−^ animals and then 24 h later infected these mice with Calb-Ag. Responding OT.I cells were subsequently analyzed in various tissues by flow cytometry. In contrast to the effect on CD4^+^ T-cells, Dectin-1 deficiency had no effect on the relative frequency of antigen-specific CD8^+^ T-cells in the mLN (Figure 2a). Moreover, unlike the CD4^+^ T-cell response, Dectin-1 deficiency did not affect division (Figure 2b) or activation (Figure 2c) of OT.I cells in any tissue analyzed. Thus, these data show that Dectin-1 controls fungal-specific CD4^+^, but not CD8^+^, T-cell proliferation to the GI tract.

### Dectin-2 does not control CD4^+^ T-cell function in the GI tract

**Figure 3:**
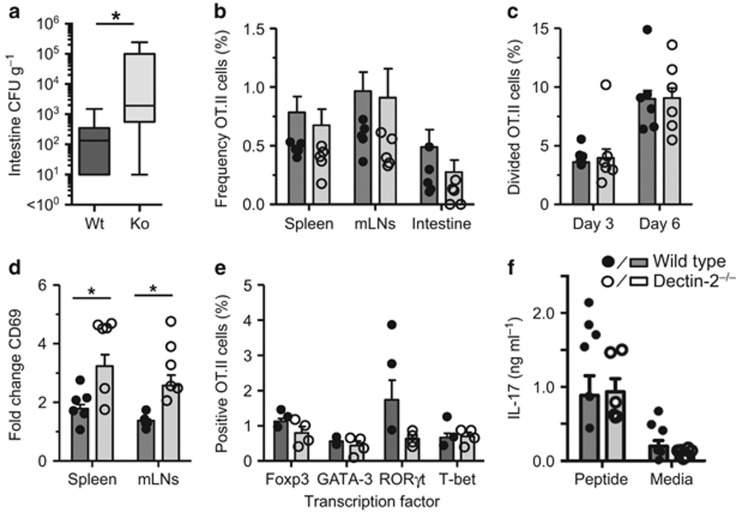
Dectin-2 is not required for antigen-specific CD4^+^ T-cell responses in the GI tract. WT (filled bars/circles) and Dectin-2 KO (light gray bars/circles) mice were analyzed as described for Dectin-1 KO mice for (**a**) intestinal fungal burdens (WT *n*=13, KO *n*=12), (**b**) frequency of OT.II cells (WT *n*=11, KO *n*=10), (**c**) division (WT *n*=12, KO *n*=10), (**d**) activation (WT *n*=11, KO *n*=10) and (**e, f**) polarization of OT.II cells in the mLN (WT *n*=6, KO *n*=4) at day 3 post infection. Bar charts show pooled data from three experiments; overlaid dot plot shows a representative experiment. *P<0.05.

Like Dectin-1, Dectin-2 has been shown to have a non-redundant role during systemic candidiasis *in vivo*,^6^ so we assessed whether absence of this receptor would also affect CD4^+^ T-cell responses during infection. We found significantly enhanced fungal burdens in the small intestine at 72 h post infection in the Dectin-2^−/−^ mice compared with WT (Figure 3a), similar to the Dectin-1 KO animals. However, unlike the Dectin-1 KO animals, we did not find any reductions in the frequency or proliferation of antigen-specific CD4^+^ T-cells in the GI tract or mLN of the Dectin-2^−/−^ mice (Figure 3b, c). Like the Dectin-1^−/−^ mice, CD69 expression by antigen-specific CD4^+^ T-cells was significantly enhanced in the Dectin-2^−/−^ mice (Figure 3d), suggesting that this phenotype may be related to the increased intestinal fungal burdens. We also found a reduction in the expression of RORγt in the mLN of the Dectin-2^−/−^ animals (Figure 3e), in line with published findings that Dectin-2 is required for production of Th17-polarizing cytokines by dendritic cell (DC)s;^6^ however, did not detect any reduction in IL-17 production upon *in vitro* restimulation (Figure 3f). Taken together, these data demonstrate that the loss of Dectin-2 does not alter fungal-specific CD4^+^ T-cell immunity in the GI tract. Importantly, these data also establish that high intestinal fungal burdens are not responsible for the T-cell defect observed in the Dectin-1^−/−^ mice.

### Loss of Dectin-1 does not confer global CD4^+^ T-cell defects

**Figure 4:**
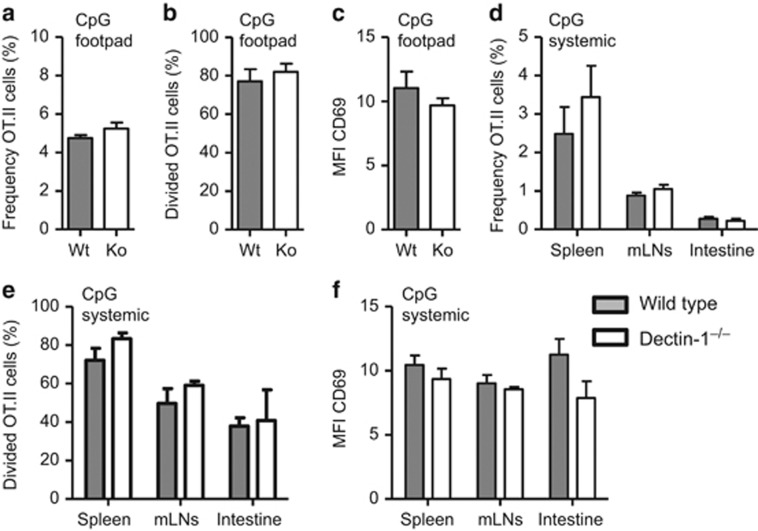
Loss of Dectin-1 does not confer global CD4^+^ T-cell defects. In total, 3 × 10^6^ OT.II T-cells were adoptively transferred into WT (filled bars/circles; *n*=5) and Dectin-1 KO (clear bars/circles; *n*=5) mice that were subsequently immunised with 3 μg ovalbumin and 2 μg CpG in the foot pad. Draining popliteal LNs were isolated at 4 days post immunization and OT.II cell (**a**) frequency, (**b**) division, and (**c**) activation analyzed as in Figure 1. (**d**–**f**) WT (*n*=4) and Dectin-1 KO (*n*=4) mice were immunised with 20 μg ovalbumin and 20 μg CpG systemically via the tail vein, and OT.II responses analyzed at 4 days post immunization as above. Data are from one experiment. CpG, oligodeoxynucleotide.

Dectin-1 has been demonstrated to have endogenous T-cell ligands^20^ and to be expressed in the corticomedullary junctions of the thymus.^21^ Therefore, we next considered the possibility that the loss of Dectin-1 was broadly affecting CD4^+^ T-cell immunity. To examine this possibility, we analyzed OT.II responses in Dectin-1 KOs immunised in the footpad with purified OVA together with the Toll-like receptor 9 agonist oligodeoxynucleotide. However, we did not find any reduction in the frequency of antigen-specific OT.II cells (Figure 4a), or their proliferation and activation in the draining LNs in these animals (Figure 4b, c).

In the absence of a global defect, we next sought to determine whether Dectin-1 was specifically required for T-cell function in the GI tract. We first attempted oral administration of Calb-Ag to test antigen-specific T-cell responses to fungi colonizing the GI tract; however, we found that this model did not sufficiently stimulate transferred OT.II cells (data not shown). Instead, we therefore immunised WT and Dectin-1 KOs systemically via the tail vein with OVA and oligodeoxynucleotide, and analyzed responding OT.II cells in the spleen and the GI tract. Similar to the subcutaneous immunisations described above, the absence of Dectin-1 had no effect on the antigen-specific CD4^+^ T-cell response (Figure 4d–f). These results therefore demonstrate that the loss of Dectin-1 does not influence T-cell immunity in response to unrelated ligands, and that this receptor is specifically required to control CD4^+^ T-cell responses in the GI tract during fungal infection.

### Loss of Dectin-1 alters DC responses in the mLN

**Figure 5:**
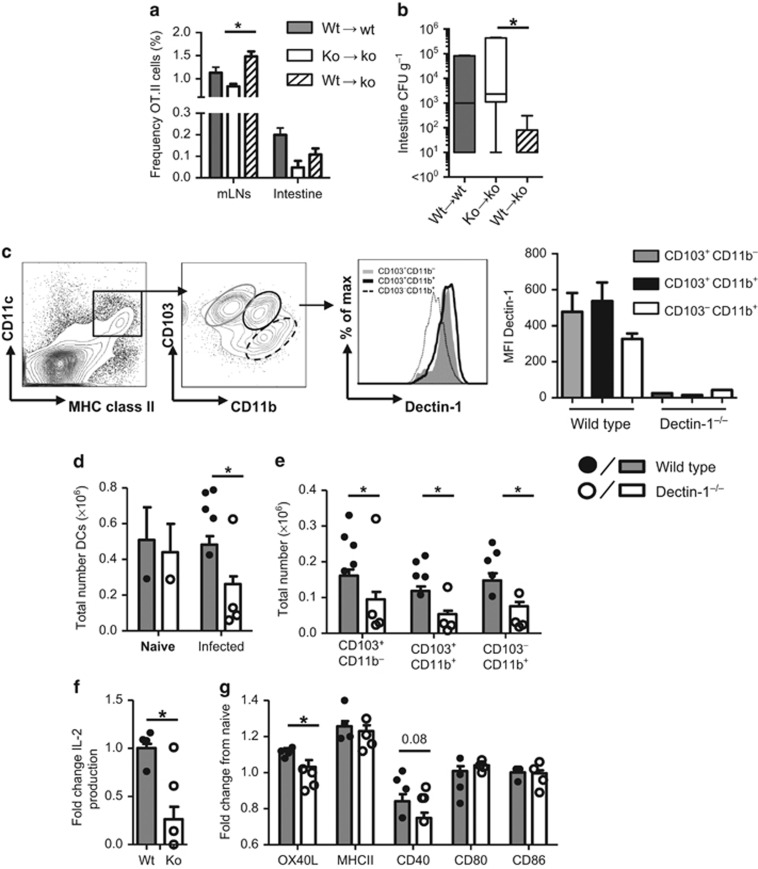
Loss of Dectin-1 causes defects in the DC response. (**a**) WT bone marrow restores the frequency of OT.II T-cells in the Dectin-1^−/−^ mLN (WT→WT *n*=7, KO→KO *n*=5, WT→KO *n*=7) and small intestine (WT→WT *n*=3, KO→KO *n*=3, WT→KO *n*=4), and also leads to a reduction in (**b**) intestinal fungal burdens (WT→WT *n*=3, KO→KO *n*=4, WT→KO *n*=7). WT→WT denotes WT animals reconstituted with WT bone marrow. WT→KO denotes KO animals reconstituted with WT bone marrow. KO→KO denotes KO animals reconstituted with KO bone marrow. (**c**) DCs in the mLN were defined as B220^−^CD11c^+^MHCII^+^ (left panel), and migratory/resident sub-populations grouped into three sub-populations based on CD103 and CD11b expression (middle panel). Dectin-1 expression was assessed by staining with 2A11^37^ and analysis by flow cytometry (right panel and graph, *n*=4). (**d**) The number of total CD11c^+^MHCII^+^ cells (infected: WT *n*=29, KO *n*=23, naive: WT *n*=4, KO *n*=4) and (**e**) the migratory/resident populations (WT *n*=19, KO *n*=16) within the mLN at 72 h post infection in WT and Dectin-1^−/−^ mice. (**f**) 4 × 10^4^ CD11c^+^ cells from WT (*n*=8) and Dectin-1^−/−^ (*n*=8) mice were purified from the mLN at 72 h post infection and cultured with 2 × 10^5^ naive OT.II T-cells and 10 μg ml^−1^ OVA_323-339_ peptide. IL-2 in the supernatants was measured 24 h later and expressed relative to the WT. (**g**) FACS analysis of the indicated co-stimulation molecules relative to naive controls at 72 h post infection by CD11c^+^MHCII^+^ cells in the mLN (*n*=12). Bar charts show pooled data (chimera/IL-2: two experiments; DC data: five experiments); overlaid dot plots show one representative experiment. *P<0.05.

We next determined whether haematopoietic or stromal components were involved in the Dectin-1 KO phenotype. For these experiments, we generated bone marrow chimeric mice and then characterized the effects of infection on OT.II responses in the GI tract in these animals. We found that reconstitution of Dectin-1-deficient mice with WT bone marrow restored the frequency of OT.II cells in the mLN and intestinal tissue (Figure 5a). Notably, there was a significant reduction in intestinal fungal burdens in animals reconstituted with WT bone marrow compared with mice reconstituted with KO bone marrow (Figure 5b).

As these results suggested that a haematopoietic component was largely responsible for the phenotype observed in the Dectin-1 KO animals, we next explored DC responses in the GI tract of these mice. For these experiments, we used flow cytometry to compare different DC sub-populations within the mLN, based on their expression of CD103 and CD11b; an approach that allows the identification of developmentally and functionally distinct DC subsets^22^ (Figure 5c). In naive WT animals, we found the highest expression of Dectin-1 on migratory CD103^+^ DC subsets in the mLN (Figure 5c). Following infection with *C. albicans*, we found a significant reduction in the number of total DCs (CD11c^+^ MHC II^+^) and the three major DC subsets in the infected Dectin-1 KO mLN, whereas no difference was found in naive animals (Figure 5d, e). Thus, these data show that absence of Dectin-1 during infection leads to abrogated DC numbers in the mLN.

### Dectin-1 is required for efficient CD4^+^ T-cell activation

Defects in T-cell co-stimulation have been previously shown to result in aberrant proliferation and activation,^23^ thus we next investigated the ability of Dectin-1^−/−^ DCs isolated from the infected mLN to activate CD4^+^ T-cells *in vitro*. To analyse this aspect of the DC response, we purified CD11c^+^ cells from infected WT and Dectin-1^−/−^ mLNs and used equivalent numbers of these cells to stimulate naive OT.II T-cells *in vitro*. We found that OT.II cells had lower levels of proliferation when co-cultured with Dectin-1^−/−^ CD11c^+^ cells compared with those cultured with WT CD11c^+^ cells (Supplementary Figure S3), mimicking what we had earlier seen *in vivo* (Figure 1i). Critically, when we measured production of IL-2 in the supernatants of our OT.II co-cultures by enzyme-linked immunosorbent assay, we found that OT.II cells co-cultured with purified Dectin-1^−/−^ CD11c^+^ cells produced significantly less IL-2 compared with T-cells co-cultured with WT CD11c^+^ cells (Figure 5f), demonstrating a defect in CD4^+^ T-cell activation by Dectin-1^−/−^ DCs. To gain further insights, we analyzed expression of co-stimulatory molecules by DCs in the mLN following infection using flow cytometry. This analysis revealed reductions in the expression of CD40 and OX40L, whereas expression of CD80, CD86, and MHC Class II remained intact (Figure 5g). Therefore, in addition to reduced numbers, loss of Dectin-1 expression by DCs in the mLN causes defects in CD4^+^ T-cell activation.

### Dectin-1 is required for CD4^+^, but not CD8^+^, T-cell viability in the GI tract

**Figure 6:**
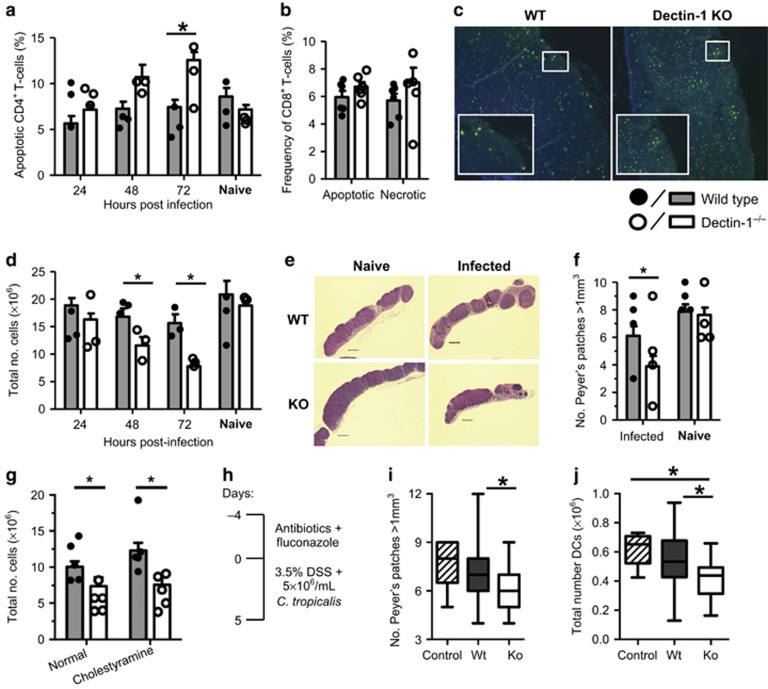
Dectin-1 is required for CD4^+^ T-cell survival. (**a**) The frequency of apoptotic CD4^+^ T-cells was measured by Annexin-V staining in mLN cell suspensions at the indicated time points post infection (WT *n*=9, KO *n*=9 at each time point). (**b**) CD8^+^ T-cell apoptosis and necrosis was measured by Annexin-V/7-AAD staining in mLN cell suspensions at 72 h post infection (*n*=6). (**c**) TUNEL staining performed on paraformaldehyde-fixed mLNs from infected WT and KO mice 72 h post infection; photo taken at 5 × magnification, insert at 40 × magnification. Blue=DAPI, green=apoptotic cells. (**d**) The total number of recoverable cells from the mLN (WT *n*=9, KO *n*=9 at each time point). (**e**) H&E staining of mLNs at 72 h post infection compared with naive controls (scale bars are 500 μm). (**f**) The total number of visible (>1 mm^3^) Peyer's patches (infected *n*=9, naive *n*=8) in WT and Dectin-1^−/−^ mice at 72 h post infection. (**g**) The total number of recoverable cells from the mLN of infected WT (*n*=10) and KO (*n*=9) mice at 3 days post infection while maintained on cholestyramine diets, as described in Figure 1. (**h**) Schematic of the colitis model where co-housed WT and Dectin-1 KO animals were maintained on water supplemented with penicillin, streptomycin, and fluconazole for 4 days, and then switched to water containing 3.5% DSS and *C. tropicalis* for 5 days. The number of (**i**) Peyer's patches and (**j**) number of DCs (CD11c^+^MHCII^+^) isolated from mLNs from WT (*n*=20) and Dectin-1 KO (*n*=16) mice at 5 days post DSS exposure was compared with *Candida* only WT animals (control, *n*=6). Bar charts show pooled data (2–5 experiments); overlaid dot plots show one representative experiment. *P<0.05.

As we found defects in DC number and co-stimulation we hypothesized that this would impact on CD4^+^ T-cell survival, as sufficient T-cell priming is required for initiation of pro-survival programs.^24^ Attempts to analyse T-cell survival *in vitro* was complicated by high background levels of apoptosis that occurred in isolated lymphocytes, following their purification and culture. Therefore, we measured the frequency of apoptotic endogenous CD4^+^ T-cells *in vivo* using Annexin-V. In naive mice, we observed an equivalent level of apoptotic CD4^+^ T-cells in the mLNs of the WT and Dectin-1^−/−^ animals (Figure 6a). In contrast, following infection, there was a time-dependent increase in the frequency of apoptotic CD4^+^ T-cells in the mLN of Dectin-1^−/−^ mice (Figure 6a and Supplementary Figure S4). Adoptively transferred OT.II cells showed similarly increased apoptosis in infected Dectin-1^−/−^ mice (Supplementary Figure S5). Dectin-1 deficiency had no effect on the viability of CD8^+^ T-cells (Figure 6b) or myeloid populations, including DCs, but we did observe increased apoptosis within the B-cell population (Supplementary Figure S5). Enhanced apoptosis in the Dectin-1^−/−^ mLN could also be qualitatively observed using terminal deoxynucleotidyl transferase dUTP nick end labeling staining (Figure 6c). In addition to this increased apoptosis, infection caused a substantial reduction in the cellularity of the mLN in the Dectin-1^−/−^ mice over time (Figure 6d), which became macroscopically smaller during infection (Figure 6e). Decreased cellularity was only observed in the mLN and not in other tissues, such as the renal LN (Supplementary Figure S6). The loss in cellularity affected all cell populations (Supplementary Figure S6). It is unclear whether this phenotype is directly related to increased CD4^+^ T-cells and B-cell apoptosis, since we did not find any significant reductions in the frequencies of individual lymphocyte populations after infection in the Dectin-1^−/−^ mLN (Supplementary Figure S6). Notably, the loss in cellularity was not restricted to the mLN, as there were also less-visible Peyer's patches in the Dectin-1^−/−^ small intestine (Figure 6f). These effects only occurred during infection, as in naive animals these tissues were equivalent to those of WT mice (Figure 6d–f). Moreover, these defects were specific to Dectin-1, as no reduction in mLN cellularity or number of Peyer's patches occurred during infection in the Dectin-2^−/−^ mice (Supplementary Figure S7). There was also no effect on mLN cellularity when the Dectin-1 KO animals were maintained on cholestyramine diet (Figure 6g), supporting our previous observation (see Figure 1c and e) that increased bile acids in the GI tract are not responsible for the phenotype in these tissues during infection. Thus, our data reveal a specific function for Dectin-1 in the maintenance of gut-associated lymphoid organs during fungal infection.

### Dectin-1 is required for maintenance of intestinal lymphoid tissues during colitis

Our data have shown that Dectin-1 has a crucial role in the maintenance of GI-lymphoid tissue following systemic fungal infection. To gain insight into the broader ramifications of these observations, we explored the possibility that Dectin-1 functions were similarly required during a breach of intestinal barrier function, such as occurs during colitis.^8^ Notably, enhanced colitis was not found in Dectin-1 KO mice in the absence of pathogenic fungi,^8^ nor did Dectin-1 KO mice exhibit abnormalities in controlling *Candida* when the intestinal barrier is intact.^14^ Thus, to explore a role for Dectin-1 during disruption of the mucosal barrier, we used a modified version of our murine model of colitis,^8^ where co-housed WT and Dectin-1^−/−^ mice were exposed to *C. tropicalis* and DSS simultaneously via their drinking water for 5 days (Figure 6h). *C. tropicalis* was chosen for these experiments based on recent data, which showed that this species specifically enhanced colitis in Dectin-1^−/−^ mice.^8^ Exposure of mice to DSS and *C. tropicalis* induced typical signs of colitis including colon shortening, which was more pronounced in the Dectin-1^−/−^ mice (Supplementary Figure S8a) as we had shown previously.^8^ In addition, the Dectin-1 KO animals fed on DSS and *C. tropicalis* had significant reductions in the number of Peyer's patches, which was not observed in WT mice (Figure 6i). In this model, the cellularity of the mLN was highly variable, and we could not detect differences in the size of this tissue in the Dectin-1 KO mice (Supplementary Figure S8b). However, we could detect reproducible reductions in the number of DCs in the mLN (Figure 6j), similar to what we had observed during systemic *C. albicans* infection. In summary, our data demonstrate that Dectin-1 is important for intestinal immune responses to pathogenic fungal flora following disruption of the mucosal barrier.

### WT DCs restore CD4^+^ T-cell responses and mLN cellularity in Dectin-1 KO mice

**Figure 7:**
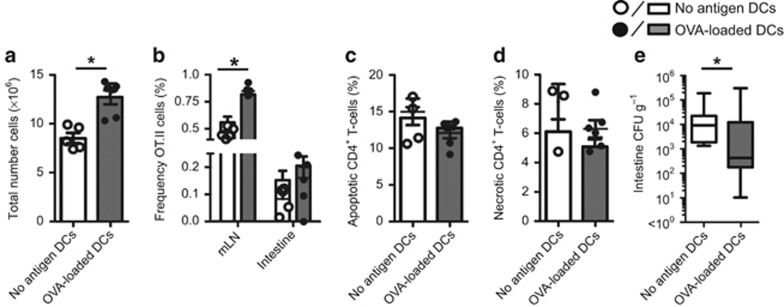
Transfer of WT DCs restores T-cell responses and mLN cellularity in Dectin-1 KO mice. CD11c^+^ cells from the mLN of naive WT mice were pre-loaded with OVA or left untreated, and injected intraperitoneally into Dectin-1 KO recipients. OT.II cells were then transferred and Calb-Ag used to infected mice, as for previous experiments. At 72 h post infection, animals were analyzed for (**a**) total number cells in the mLN, (**b**) frequency of OT.II cells, (**c**) CD4^+^ T-cell apoptosis and (**d**) necrosis, and (**e**) intestine fungal burdens. All data are pooled from two independent experiments; overlaid dot plots show one representative experiment. *P<0.05.

We next sought to understand the link between the aberrant CD4^+^ T-cell response and mLN hypocellularity in the infected Dectin-1 KO mice. The alteration in the number and function of DCs in the mLN, and our ability to restore T-cell responses by reconstituting Dectin-1 KO mice with WT bone marrow (Figure 5), suggested that defective DC responses were responsible for the observed phenotype. To test this hypothesis, we injected Dectin-1 KO animals intraperitoneally with CD11c^+^ cells purified from the mLN of naive WT mice, which were either pre-loaded with OVA or left untreated. We found this method of DC transfer allowed DCs to migrate into the infected mLN, as we could track labeled DCs to this site post transfer (Supplementary Figure S9a). Following DC transfer, these animals were subsequently given OT.II cells and infected with Calb-Ag, as before. We found that treatment with OVA-loaded DCs significantly restored the cellularity of the mLN (Figure 7a), compared with infected KO mice that received unloaded WT DCs. Moreover, OVA-loaded DC-treated mice also had an increased frequency of OT.II cells in the mLN and the small intestine (Figure 7b). Interestingly, treatment with OVA-loaded DCs only slightly reduced CD4^+^ T-cell apoptosis in the mLN (Figure 7c and d), suggesting that there are other contributing factors for this particular phenotype. Importantly, we found that treatment with OVA-loaded DCs significantly reduced fungal burdens in the small intestine (Figure 7e). We next tested whether the defect in Dectin-1 DC function was simply a lack of activation, by pre-stimulating antigen-loaded Dectin-1 KO DCs with LPS prior to adoptive transfer. However, we found that KO mice transferred with stimulated KO DCs still had a significantly reduced number of cells in their mLN, compared with the mLN of KO mice that had received WT DCs stimulated in the same way (Supplementary Figure S9b). Thus, these data show that Dectin-1^+^ DCs control mLN cellularity and antigen-specific CD4^+^ T-cell responses in the GI tract during systemic candidiasis.

## Discussion

The far-reaching influence of commensal GI microbes on immune development and maintenance has led to intense interest in the regulation of intestinal barrier function and mucosal immunity. The influence of commensal fungi in the GI tract, in particular, is only beginning to be explored. Herein, we describe a specific role for Dectin-1 in controlling CD4^+^, but not CD8^+^, T-cell responses and survival during fungal infection in the GI tract. Moreover, we demonstrate how loss of this receptor also results in substantial reductions in the cellularity of the GI-associated lymphoid organs during systemic infection and in colitis. This reduction in mLN cellularity affected multiple cell types without altering cellular frequency, whereas we only observed increased apoptosis in the CD4^+^ T-cell and B-cell compartments. It is possible that the increased T and B-cells apoptosis are interlinked, as B-cell survival can require T-cell help.^25^ Thus, further investigation is required to understand how loss of Dectin-1 can result in the rapid reduction of mLN cellularity during systemic candidiasis, and how this phenotype relates to the defects in CD4^+^ T-cell responses. Finally, we show that the loss in mLN cellularity stems from defects in the mLN DC compartment, as transfer of Dectin-1^+^ DCs into KO mice restored fungal-specific CD4^+^ T-cell responses and mLN cellularity during infection, although it did not restore the effect on CD4^+^ T-cell apoptosis.

Our data reveal that signaling through Dectin-1 differentially controls the ability of DCs to induce CD4^+^, but not CD8^+^, T-cell responses during infection *in vivo*. In the GI tract, DCs can be grouped into three sub-populations based on their expression of CD103 (integrin αE) and/or CD11b. CD103^+^ DCs in the mLN are thought to denote cells that have migrated from the intestine to the mLN, whereas CD11b^+^CD103^-^ DCs in the mLN are thought to be non-migratory ‘resident' DCs.^22^ However, recent reports showing trafficking of intestinal CD103^-^ DCs to the mLN^26^ indicates that DC movement in the GI tract is more complex than previously appreciated. In the infected mice, the loss of Dectin-1 resulted in significant reductions in the number of both migratory CD103^+^ and resident CD103^−^ DCs in the mLN. In contrast, in naive animals, there were no differences in these DC populations. Moreover, DCs isolated from the mLN of infected mice had a poor ability to co-stimulate CD4^+^ T-cells *in vitro*, indicating that Dectin-1 is involved in multiple complex pathways of DC activation in the GI tract during fungal infection. As we could not find any differences in these cell populations in naive mice, this indicates that Dectin-1 is important for the maintenance of DC populations during pathogenic fungal infection but is unaffected by commensal microbes, including fungi, in the gut.^8^ Indeed, these data are consistent with our previous observation showing that Dectin-1 expression is not detected on the luminal-facing side of the intestinal barrier,^21^ and that this receptor is not involved in controlling commensal populations of *C. albicans* in the GI tract.^14^

CD103^−^ and CD103^+^ DCs are thought to have blood-derived precursors.^27, 28^ In line with this, we found that reconstitution of Dectin-1-deficient mice with WT bone marrow restored CD4^+^ T-cell responses and the control of fungal growth in the GI tract. In further experiments, we show that this restoration is specifically due to DCs, as transfer of these cells alone was sufficient to rescue the phenotype of the Dectin-1 KO animals during infection. These results may stem from aberrant trafficking of the Dectin-1 KO DCs, which was not determined in this study. Although DC subsets in the GI tract may have a common progenitor, they have been shown to mediate distinct functions. For example, CD103^+^CD11b^+^ DCs were recently shown to be important drivers of Th17 immunity in the GI tract through IL-6 production,^29^ whereas the specific absence of CD103^+^CD11b^−^ DCs had no effect on Th17 polarization.^30^ In addition to independent functions, DC subsets in the mLN also have shared functions; the expression of gut-homing marker CCR9 on T_reg_ cells in the mLN was shown to be severely reduced in the absence of both these subsets, but was intact in singly subset deficient mice.^31^ Thus, our data suggest that in the GI tract, DCs require Dectin-1 to promote downstream CD4^+^ T-cell responses during fungal infection. Interestingly, although Dectin-1^+^ DCs could rescue the KO animals, they were not sufficient to fully restore CD4^+^ T-cell survival during infection, an observation that requires further exploration.

Dectin-1 is required for protecting the intestinal barrier from invading fungi, as Dectin-1-deficient mice are more susceptible to colitis in the presence of pathogenic fungi.^8^ As occurred during systemic infection, we show here that a breach in barrier integrity in the presence of pathogenic fungi led to reductions of DCs in the mLN. Moreover, this treatment caused significant reductions in the number of Peyer's patches in the Dectin-1 KO small intestine. Recruitment of DCs to the mLN are crucial for the generation of mucosal T-cell responses,^26, 29, 30, 31^ and disruption of intestinal DC migration has been linked with poor control of bacterial and parasitic infections.^32, 33^ Therefore, our observations reveal a novel role for Dectin-1 in controlling fungal-mediated inflammation during colitis.^8^

This gut-tropic function of Dectin-1 has broad implications for our understanding of the control of fungi in the GI tract. Humans with a polymorphism in Dectin-1, which renders them essentially deficient for this receptor,^34^ have higher GI colonization levels of *Candida* compared with control patients, increasing the risk of systemic infection following ablative treatment for organ transplant.^35^ Moreover, disruption of GI barrier integrity is thought to be a common origin of infection in patients with systemic candidiasis.^11, 12^ Our data reveal one of the mechanisms in which Dectin-1 contributes to protection of the intestinal tissues against invasion by pathogenic fungi, and how these mechanisms are disrupted in the absence of this receptor.

In conclusion, Dectin-1 is specifically required for CD4^+^ T-cell responses in the murine GI tract in response to fungal challenge. This function of Dectin-1 has important implications for understanding how fungi influence adaptive immunity at this mucosal site, and the immune mechanisms required to control these pathogens during disease.

## Methods

**Mice.** Eight to 12-week-old female C57BL/6, Dectin-1^−/−^,^14^ Dectin-2^−/−^,^6^ OT.I, and OT.II mice were bred and maintained in individually ventilated cages at the Medical Research Facility at the University of Aberdeen. Mice were co-housed for all experiments. All experimentation conformed to the terms and conditions of United Kingdom Home Office license 60/4007 for research on animals and the University ethical committee.

**OT.II adoptive transfers.** CD4^+^ cells were negatively purified (Miltenyi Biotech, Surrey, UK) from lymph nodes and spleens isolated from OT.II donor mice. Purified CD4^+^ cells were stained with 5 μM carboxyfluorescein succinimidyl ester (Invitrogen, Paisley, UK) and 3 × 10^6^ cells were then injected intravenously into gender-matched recipient mice.

**DC adoptive transfers.** CD11c^+^ cells were positively purified (Miltenyi Biotech) from the mLN of naive WT mice, and resulting cells plated out at 2.5 × 10^5^ ml^−1^ in Roswell Park Memorial Institute (RPMI) (Invitrogen) containing GlutaMAX and 25 mM 4-(2-hydroxyethyl)-1-piperazineethanesulfonic acid, further supplemented with 10% fetal calf serum, penicillin (100 units ml^−1^), streptomycin (100 units ml^−1^) and 50 μM β-mercaptoethanol. Some cells were further supplemented with 10 μg ml^−1^ OVA (EndoGrade, Hyglos, Munich, Germany). DCs were incubated at 37 °C for 18 h, washed in 1 × phosphate-buffered saline and 5 × 10^5^ cells injected intraperitoneally into Dectin-1 KO-recipient animals.

**Systemic candidiasis model.** Co-housed animals were injected intravenously with 2 × 10^5^ CFU of *C. albicans* strain Calb-Ag,^19^ which was grown in yeast extract peptone medium. Fungal burdens were determined by serial dilution on yeast extract peptone agar supplemented with 100 μg ml^−1^ Gentamicin and 10 μg ml^−1^ Vancomycin and incubation at 37 °C for 24 h.

**Flow cytometry.** Antibodies used in this study were CD3-APC (145-2C11), CD4-APC H7 (GK1.5), Vα2-PE (B20.1), CD45.1-PerCP Cy5.5 (A20), CD69-AlexaFluor700 (H1.2F3), CD8-APC (53.6-7), CD11c-BV421 (HL3), MHC Class II-FITC (2G9), α4β7-APC (DATK32), CD103-biotin (M290), CD11b-PE Cy7 (M1/70), CD80-V450 (16-10A1), CD86-AF700 (GL1), OX40L-PE (RM134L), CD40-PE (3/23) purchased from BD Biosciences (Oxford, UK), and Foxp3-CF594 (MF23), GATA-3-eFluor710 (TWAJ), RORγt-APC (B2D), T-bet-PE Cy7 (eBio4B10), CCR9-PE Cy7 (CW-1.2) purchased from eBioscience (Hatfield, UK). Staining was performed in 1 × phosphate-buffered saline supplemented with 2% fetal calf serum and 2 mM sodium azide, and anti-CD16/32 (24G2). Intracellular staining for transcription factors was performed using the Foxp3 staining kit from eBioscience, as per manufacturer's instructions. Samples were acquired on the fluorescence-activated cell sorting LSR II cytometer (BD Biosciences). FlowJo software (Tree Star, Ashland, OR) was used for analysis.

**Generation of bone marrow chimeric mice.** Six to 8-week-old recipient mice were irradiated with two 500 rad doses, rested overnight, and prepared bone marrow injected intravenously 24 h after the first irradiation. Bone marrow isolated from femurs of gender-matched donor animals was washed and resuspended in 1 × phosphate-buffered saline for injection, with each recipient receiving 2 × 10^6^ bone marrow cells. Chimeric status of mice was confirmed with a blood sample 8–10 weeks following irradiations.

**Quantitative PCR.** RNA was isolated using TRI Reagent (Applied Biosystems, Paisley, UK) as per manufacturer's instructions. Synthesis of complementary DNA was performed on 1 μg isolated RNA using the SuperScript III First-Strand SuperMix from Invitrogen as per manufacturer's instructions, and subsequently used as a template in qPCR reactions using the SYBR green master mix (Applied Biosystems). All reactions were performed using the LightCycler480 (Roche, West Sussex, UK). The thermal cycle used contained an initial denaturing step of 95 °C for 5 min, followed by 40–50 cycles of 95 °C for 15 s and 60 °C for 1 min. Fold change in gene expression levels were derived using the ΔΔCt method, where ΔΔCt=(Ct Infected Target−Ct Infected Housekeeping)−(Ct Naïve Target−Ct Naive Housekeeping). Glyceraldehyde 3-phosphate dehydrogenase was used as the house-keeping gene in all cases. Primer sequences: glyceraldehyde 3-phosphate dehydrogenase: 5′-CCAGGTTGTCTCCTGCGACTT-3′ and 5′-CCTGTTGCTGTAGCCGTATTCA-3′. Cyp7a1: 5′-AGCAACTAAACAACCTGCCAGTACTA-3′ and 5′-GTCCGGATATTCAAGGATGCA-3′. Fgf15: 5′-ACGGGCTGATTCGCTACTC-3′and 5′-TGTAGCCTAAACAGTCCATTTCCT-3′.

**Bile-acid quantification.** To quantify bile acids in the murine small intestine, contents were collected into eppendorfs, 100 μl sterile phosphate-buffered saline added and samples vortexed thoroughly. Intestine contents were spun down, and bile-acid quantification performed on the supernatants using the bile-cid colorimetric assay from diazyme as per manufacturer's instructions.

**Intestinal isolations.** To isolate lymphocytes: small intestines were opened laterally and washed out before cutting into 1–2 cm^3^ pieces. Pieces were washed twice in calcium-magnesium-free solution (25 mM sodium bicarbonate, 10 mM 4-(2-hydroxyethyl)-1-piperazineethanesulfonic acid, 2% fetal calf serum in 1 × Hank's Balanced Salt Solution (HBSS)) and then incubated with dithioerythritol solution (1 mM dithioerythritol in calcium-magnesium-free with 10% fetal bovine serum) for 30 min at 37 °C with shaking. Pieces in dithioerythritol were vortexed for 15 s and supernatants collected and stored on ice. Pieces were then incubated with 1 mM ethylenediaminetetraacetic acid in 1 × HBSS/1% RPMI for 30 min at 37 °C with shaking, and supernatants from this incubation discarded. In total, 10,000 units of Collagenase I (Worthington) in RPMI containing 1 mM CaCl_2_, 1 mM MgCl_2_, and 5% fetal bovine serum, was then added to pieces along with five glass beads (3-mm diameter, Sigma) and incubated for 60 min at 37 °C with shaking. Supernatants were collected and pooled with dithioerythritol supernatants, centrifuged, and pellets resuspended in 40% Percoll and overlaid onto 70% Percoll prior to spinning at 2,000 rpm for 20 min at 20 °C. White cells were collected at the interphase and stained for analysis by flow cytometry. To isolate DCs: small intestines prepared as above and washed thoroughly in 2% fetal calf serum /HBSS. Pieces were then added into 2 mM ethylenediaminetetraacetic acid /HBSS and incubated, with shaking, at 37 °C for 20 min. The supernatants from this incubation were discarded, pieces washed in HBSS, and fresh ethylenediaminetetraacetic acid /HBSS added and incubation at 37 °C continued for a further 20 min. The supernatants from this second ethylenediaminetetraacetic acid step were discarded, and complete RPMI supplemented with 1 mg ml^−1^ Collagenase I (Worthington) added to the pieces. Collagenase digestion was allowed to continue for a maximum of 30 min at 37 °C, vortexing thoroughly every 5–10 min. Supernatants from the digest were collected, passed through 40 μM filters, washed extensively in fluorescence-activated cell sorting buffer and stained for analysis by flow cytometry.

***In vitro* restimulation assays.** All cells were maintained in supplemented RPMI (as above) in 96-well round-bottom plates. In some experiments, 2 × 10^6^ cells (per well) isolated from lymph nodes or spleens of infected animals were restimulated with 5 μg ml^−1^ OVA_323-339_ peptide (Genscript, Piscataway, NJ) for 3–5 days at 37 °C. In other experiments, 4 × 10^4^-purified CD11c^+^ cells (using the CD11c microbeads from Miltenyi) were incubated with 2 × 10^5^ OT.II cells (per well) for 24 h at 37 °C. At the end of the incubations, IL-17 or IL-2 in the culture supernatants were analyzed by sandwich enzyme-linked immunosorbent assay using kits from eBioscience (IL-17) and BD Biosciences (IL-2).

**Colitis model.** Co-housed WT and Dectin-1^−/−^ animals were maintained on sterile water supplemented with 2 mg ml^−1^ streptomycin, 2,000 U ml^−1^ penicillin (both Invitrogen) and 0.25 mg ml^−1^ fluconazole (Enzo, Exeter, UK) for 4 days prior to start of experiments. *C. tropicalis* AM071/0287 was resuspended at 5 × 10^6^ cells ml^−1^ in sterile water supplemented with streptomycin and penicillin (as above) and 3.5% DSS (MP Biomedicals, Santa Ana, CA, 36–50 k MW) and animals were maintained on *Candida*/DSS water for 5 days. All animals were killed 5 days post colitis induction.

**Statistical analysis.** Statistical tests were performed using GraphPad Prism 5.0. Fungal burden data were analyzed using Mann–Whitney *U*-tests. Other data were analyzed using student *t*-tests. Data were considered statistically significant if *P*<0.05.
