# Supplementary material for: CD4+ T-cell survival in the GI tract requires dectin-1 during fungal infection
Source: Mucosal Immunol. 2015 Sep 9;9(2):492–502. doi: 10.1038/mi.2015.79 (PMC4677461; doi:10.1038/mi.2015.79)
Supplement: Supplementary Figures [file mi201579x1.ppt]

## Slide 1
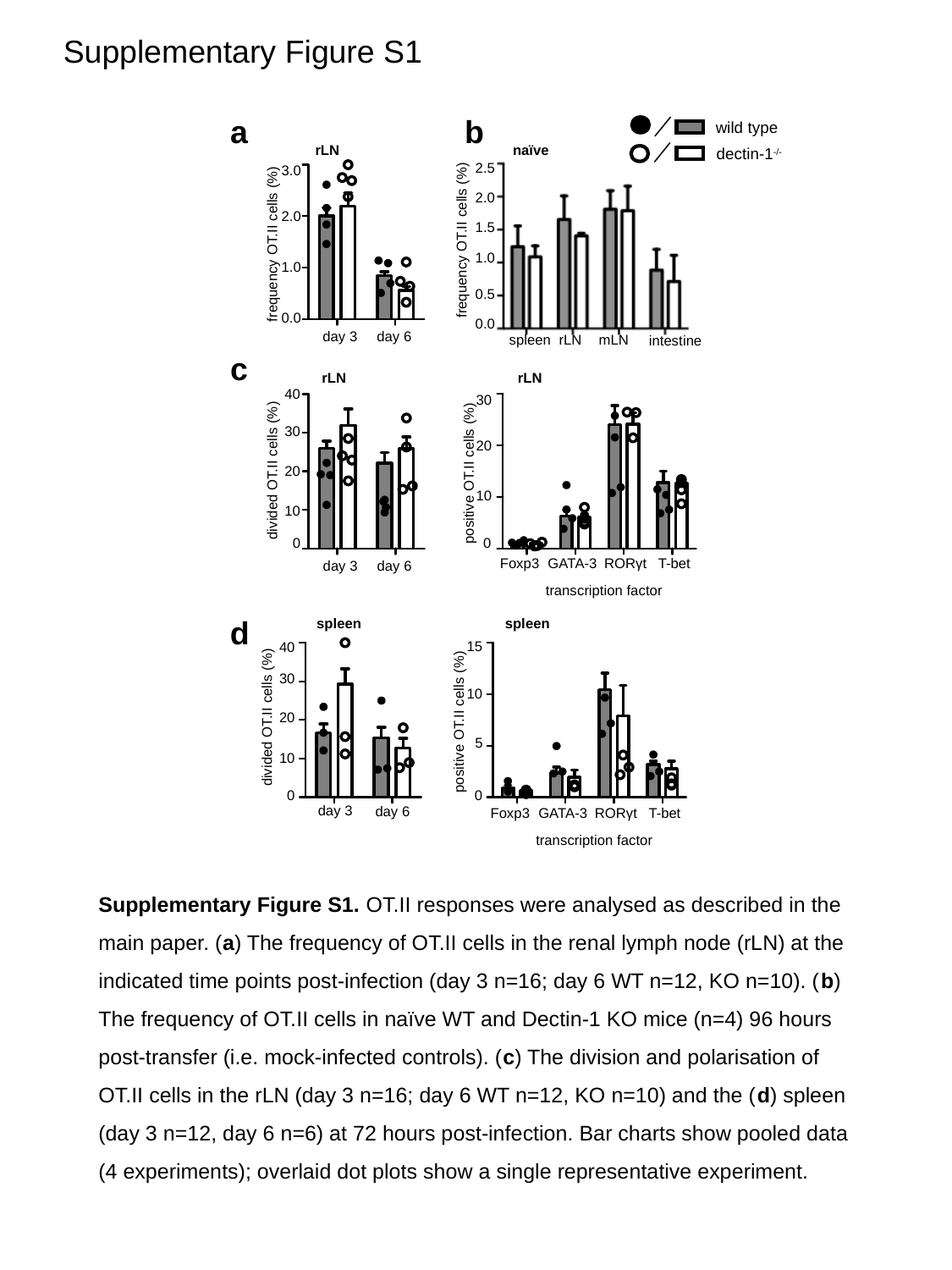

Supplementary Figure S1
a
b
wild type
dectin-1-/-
rLN
3.0
2.0
1.0
0.0
day 3
day 6
naïve
2.5
2.0
1.5
frequency OT.II cells (%)
frequency OT.II cells (%)
1.0
0.5
0.0
spleen
rLN
mLN
intestine
c
rLN
rLN
40
30
30
20
divided OT.II cells (%)
20
positive OT.II cells (%)
10
10
0
0
Foxp3
GATA-3
RORγt
T-bet
day 3
day 6
transcription factor
d
spleen
spleen
15
40
30
10
divided OT.II cells (%)
20
positive OT.II cells (%)
5
10
0
0
day 3
day 6
Foxp3
GATA-3
RORγt
T-bet
transcription factor
Supplementary Figure S1. OT.II responses were analysed as described in the main paper. (a) The frequency of OT.II cells in the renal lymph node (rLN) at the indicated time points post-infection (day 3 n=16; day 6 WT n=12, KO n=10). (b) The frequency of OT.II cells in naïve WT and Dectin-1 KO mice (n=4) 96 hours post-transfer (i.e. mock-infected controls). (c) The division and polarisation of OT.II cells in the rLN (day 3 n=16; day 6 WT n=12, KO n=10) and the (d) spleen (day 3 n=12, day 6 n=6) at 72 hours post-infection. Bar charts show pooled data (4 experiments); overlaid dot plots show a single representative experiment.

## Slide 2
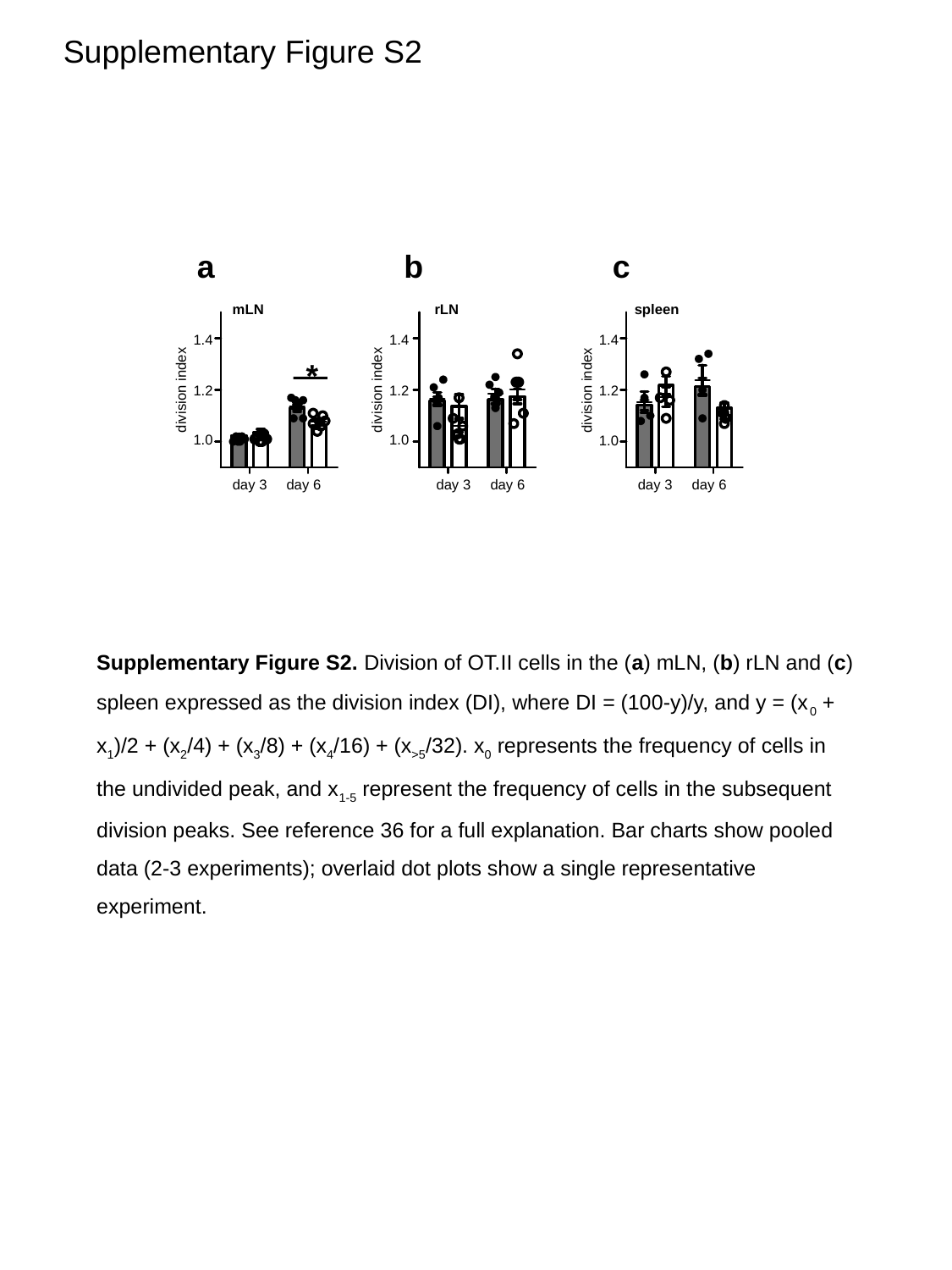

Supplementary Figure S2
a
b
c
mLN
rLN
spleen
1.4
1.4
1.4
*
division index
1.2
division index
1.2
division index
1.2
1.0
1.0
1.0
day 3
day 6
day 3
day 6
day 3
day 6
Supplementary Figure S2. Division of OT.II cells in the (a) mLN, (b) rLN and (c) spleen expressed as the division index (DI), where DI = (100-y)/y, and y = (x0 + x1)/2 + (x2/4) + (x3/8) + (x4/16) + (x>5/32). x0 represents the frequency of cells in the undivided peak, and x1-5 represent the frequency of cells in the subsequent division peaks. See reference 36 for a full explanation. Bar charts show pooled data (2-3 experiments); overlaid dot plots show a single representative experiment.

## Slide 3
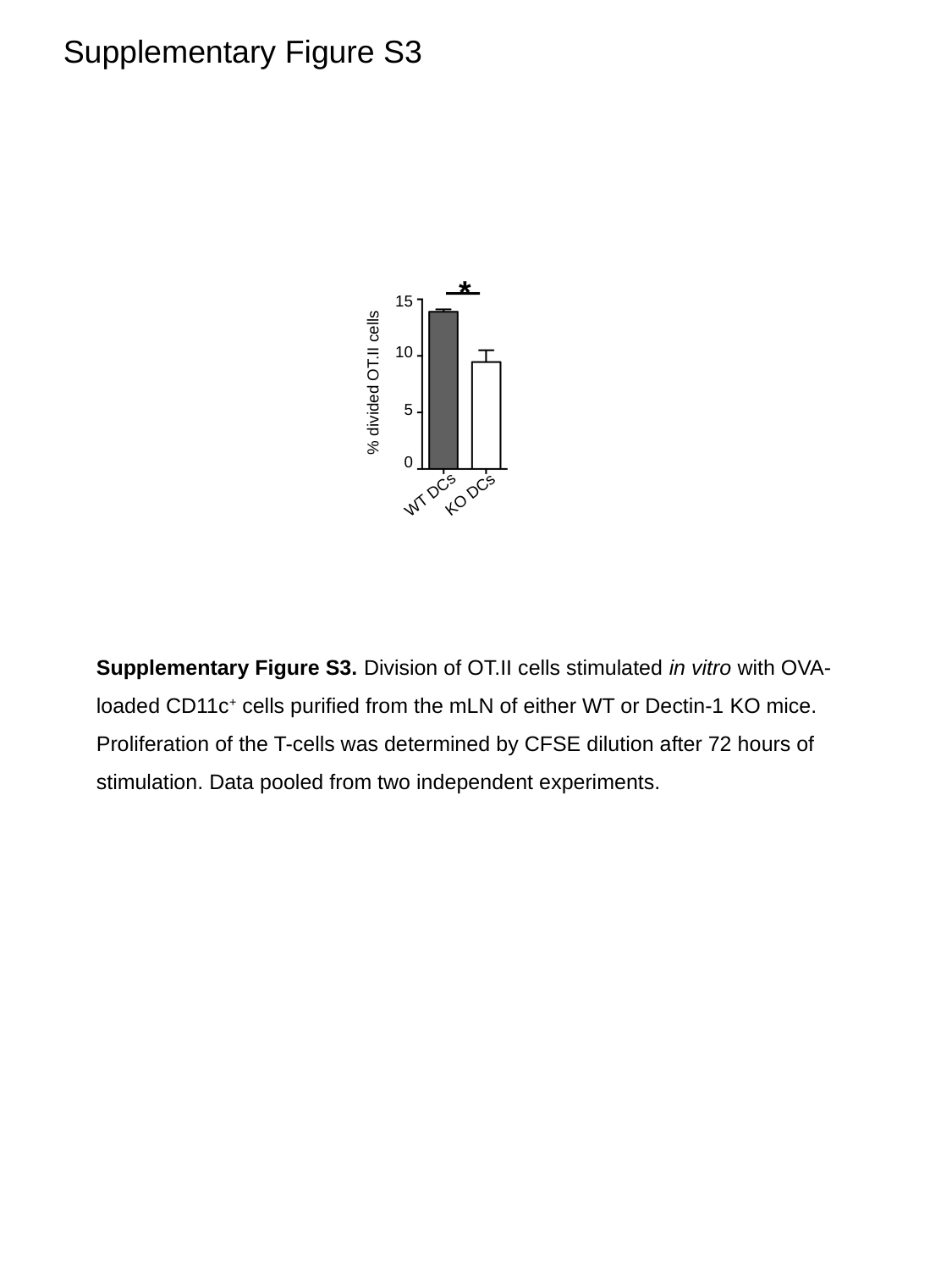

Supplementary Figure S3
*
15
10
% divided OT.II cells
5
0
KO DCs
WT DCs
Supplementary Figure S3. Division of OT.II cells stimulated in vitro with OVA-loaded CD11c+ cells purified from the mLN of either WT or Dectin-1 KO mice. Proliferation of the T-cells was determined by CFSE dilution after 72 hours of stimulation. Data pooled from two independent experiments.

## Slide 4
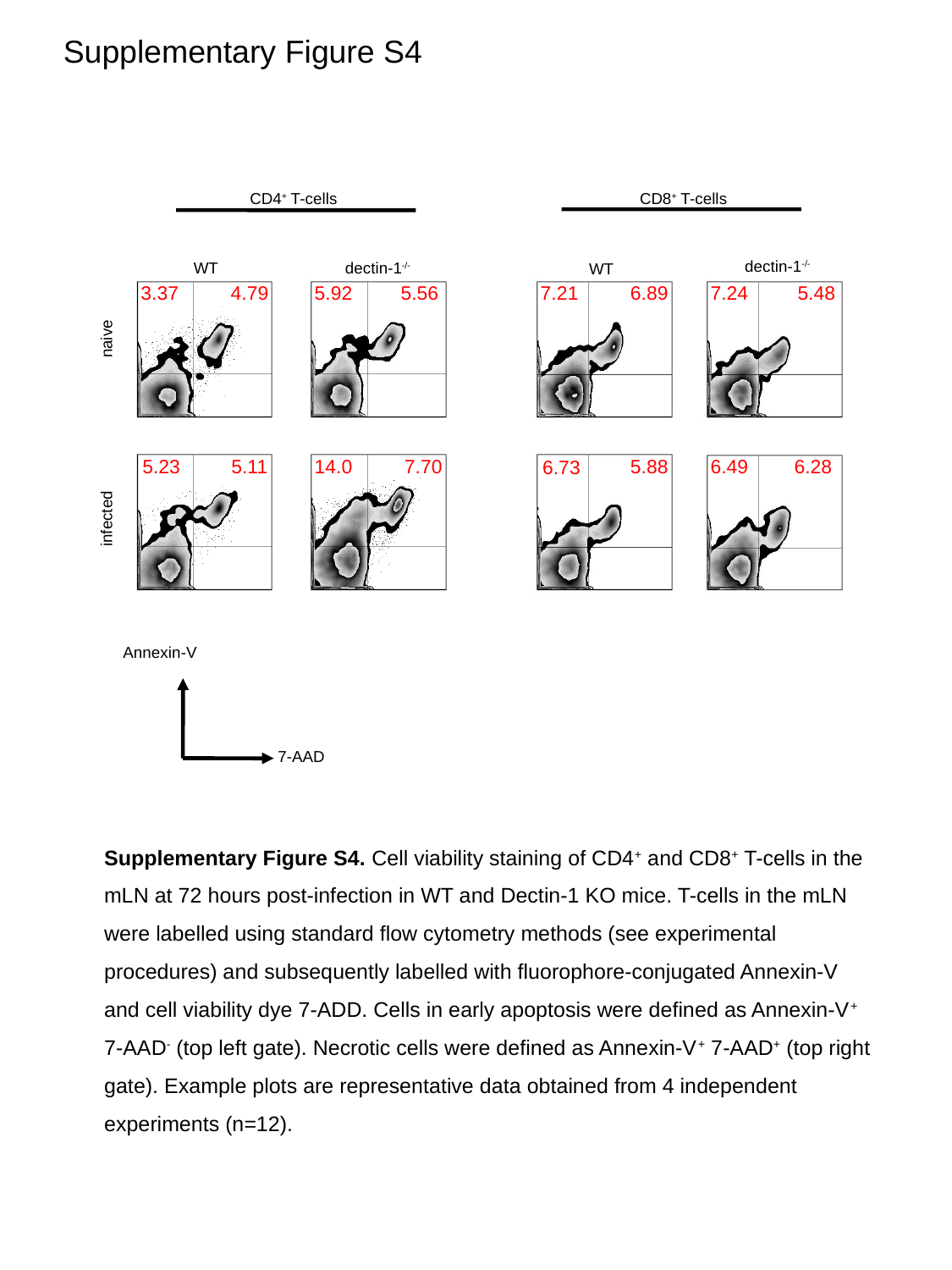

Supplementary Figure S4
CD4+ T-cells
CD8+ T-cells
dectin-1-/-
WT
dectin-1-/-
WT
3.37
4.79
5.92
5.56
7.21
6.89
7.24
5.48
naive
5.23
5.11
14.0
7.70
5.88
6.49
6.28
6.73
infected
Annexin-V
7-AAD
Supplementary Figure S4. Cell viability staining of CD4+ and CD8+ T-cells in the mLN at 72 hours post-infection in WT and Dectin-1 KO mice. T-cells in the mLN were labelled using standard flow cytometry methods (see experimental procedures) and subsequently labelled with fluorophore-conjugated Annexin-V and cell viability dye 7-ADD. Cells in early apoptosis were defined as Annexin-V+ 7-AAD- (top left gate). Necrotic cells were defined as Annexin-V+ 7-AAD+ (top right gate). Example plots are representative data obtained from 4 independent experiments (n=12).

## Slide 5
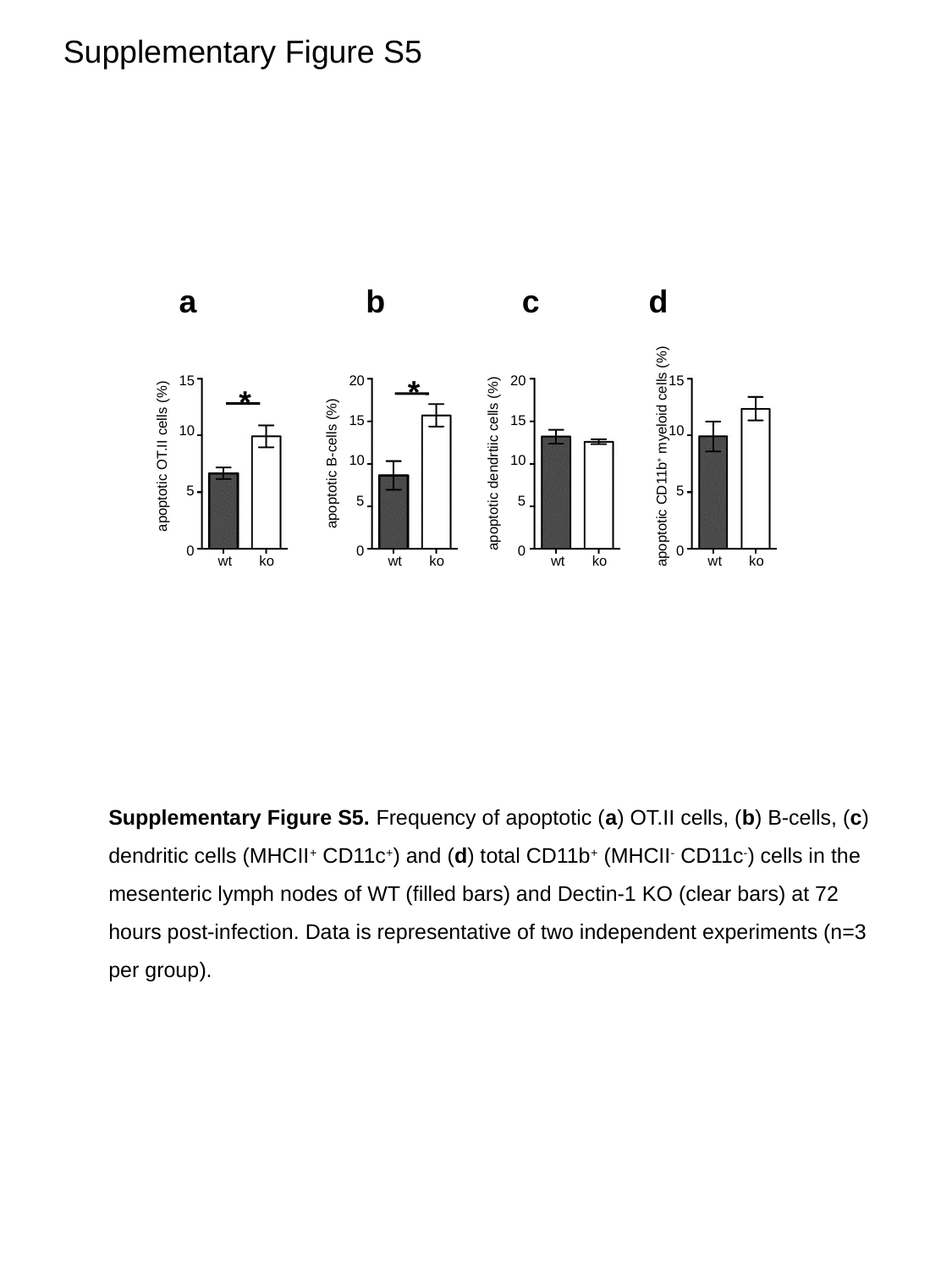

Supplementary Figure S5
a
b
c
d
15
20
*
20
15
*
15
15
10
10
apoptotic CD11b+ myeloid cells (%)
apoptotic OT.II cells (%)
10
10
apoptotic B-cells (%)
apoptotic dendrtiic cells (%)
5
5
5
5
0
0
0
0
wt
ko
wt
ko
wt
ko
wt
ko
Supplementary Figure S5. Frequency of apoptotic (a) OT.II cells, (b) B-cells, (c) dendritic cells (MHCII+ CD11c+) and (d) total CD11b+ (MHCII- CD11c-) cells in the mesenteric lymph nodes of WT (filled bars) and Dectin-1 KO (clear bars) at 72 hours post-infection. Data is representative of two independent experiments (n=3 per group).

## Slide 6
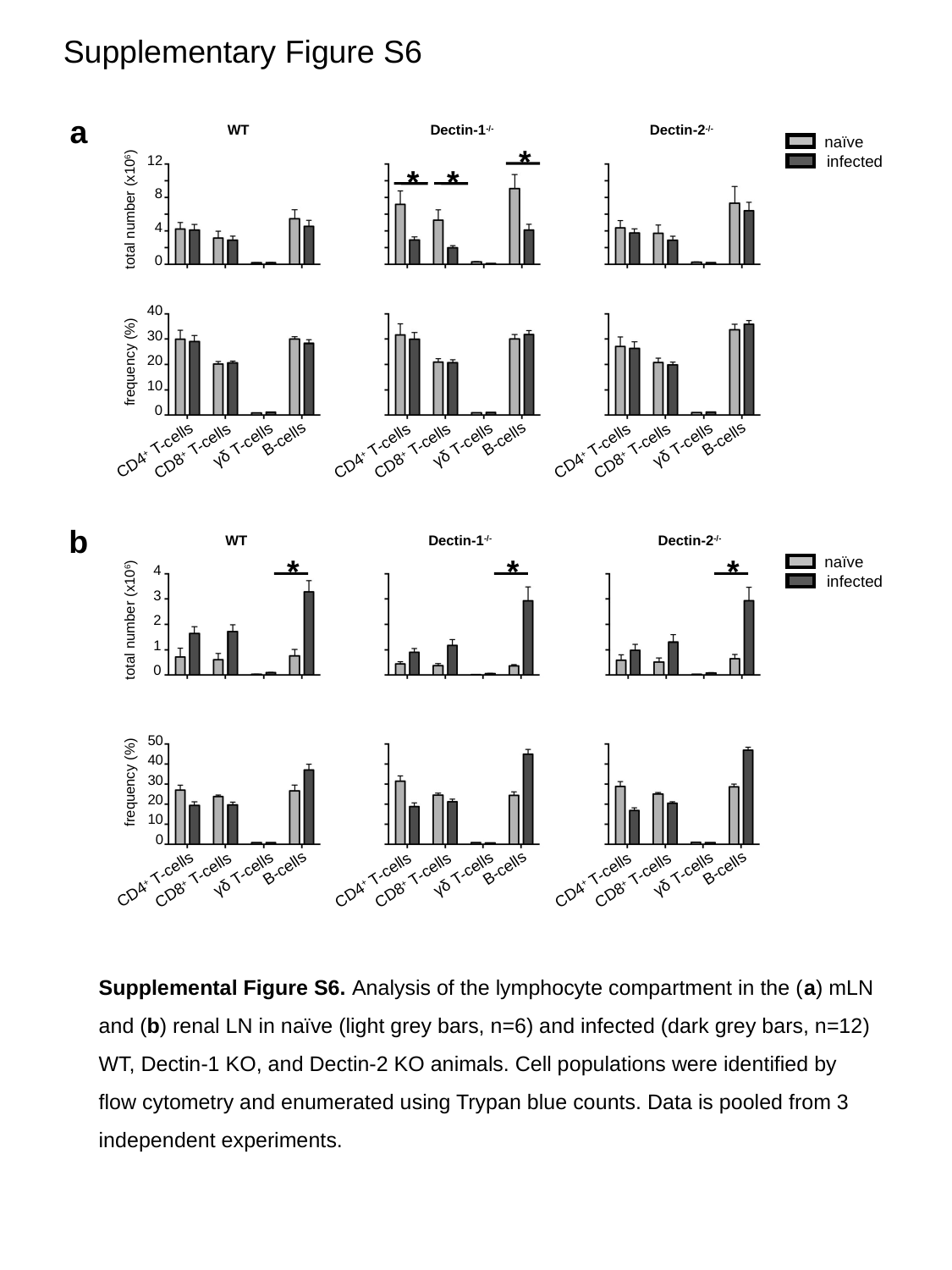

Supplementary Figure S6
a
WT
Dectin-1-/-
Dectin-2-/-
naïve
infected
*
12
*
*
8
total number (x106)
4
0
40
30
20
frequency (%)
10
0
B-cells
B-cells
B-cells
γδ T-cells
γδ T-cells
γδ T-cells
CD4+ T-cells
CD4+ T-cells
CD4+ T-cells
CD8+ T-cells
CD8+ T-cells
CD8+ T-cells
b
WT
Dectin-1-/-
Dectin-2-/-
*
*
*
naïve
infected
4
3
2
total number (x106)
1
0
50
40
30
frequency (%)
20
10
0
B-cells
B-cells
B-cells
γδ T-cells
γδ T-cells
γδ T-cells
CD4+ T-cells
CD4+ T-cells
CD4+ T-cells
CD8+ T-cells
CD8+ T-cells
CD8+ T-cells
Supplemental Figure S6. Analysis of the lymphocyte compartment in the (a) mLN and (b) renal LN in naïve (light grey bars, n=6) and infected (dark grey bars, n=12) WT, Dectin-1 KO, and Dectin-2 KO animals. Cell populations were identified by flow cytometry and enumerated using Trypan blue counts. Data is pooled from 3 independent experiments.

## Slide 7
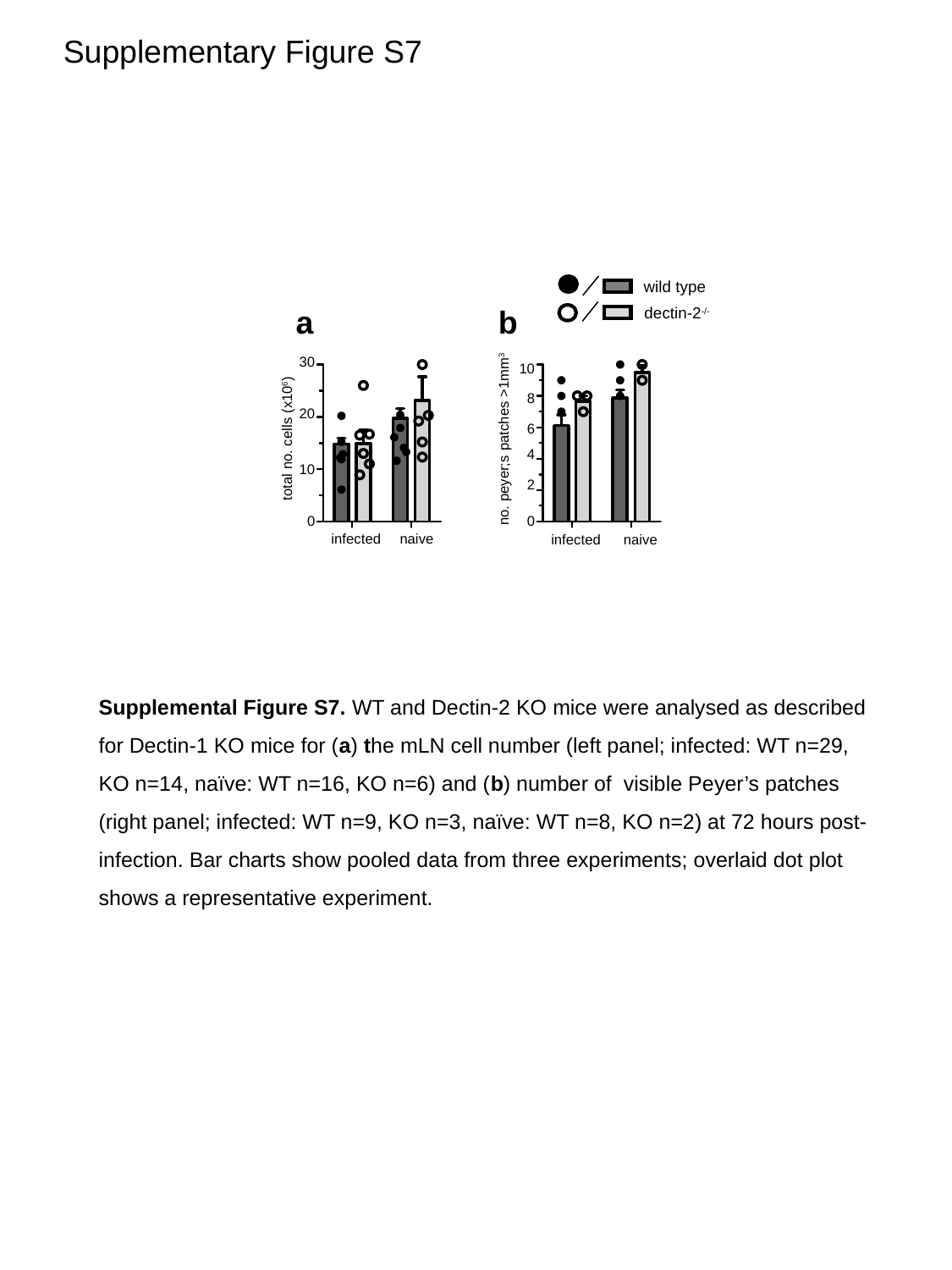

Supplementary Figure S7
wild type
dectin-2-/-
a
b
10
8
6
no. peyer;s patches >1mm3
4
2
0
infected
naive
30
20
total no. cells (x106)
10
0
infected
naive
Supplemental Figure S7. WT and Dectin-2 KO mice were analysed as described for Dectin-1 KO mice for (a) the mLN cell number (left panel; infected: WT n=29, KO n=14, naïve: WT n=16, KO n=6) and (b) number of visible Peyer’s patches (right panel; infected: WT n=9, KO n=3, naïve: WT n=8, KO n=2) at 72 hours post-infection. Bar charts show pooled data from three experiments; overlaid dot plot shows a representative experiment.

## Slide 8
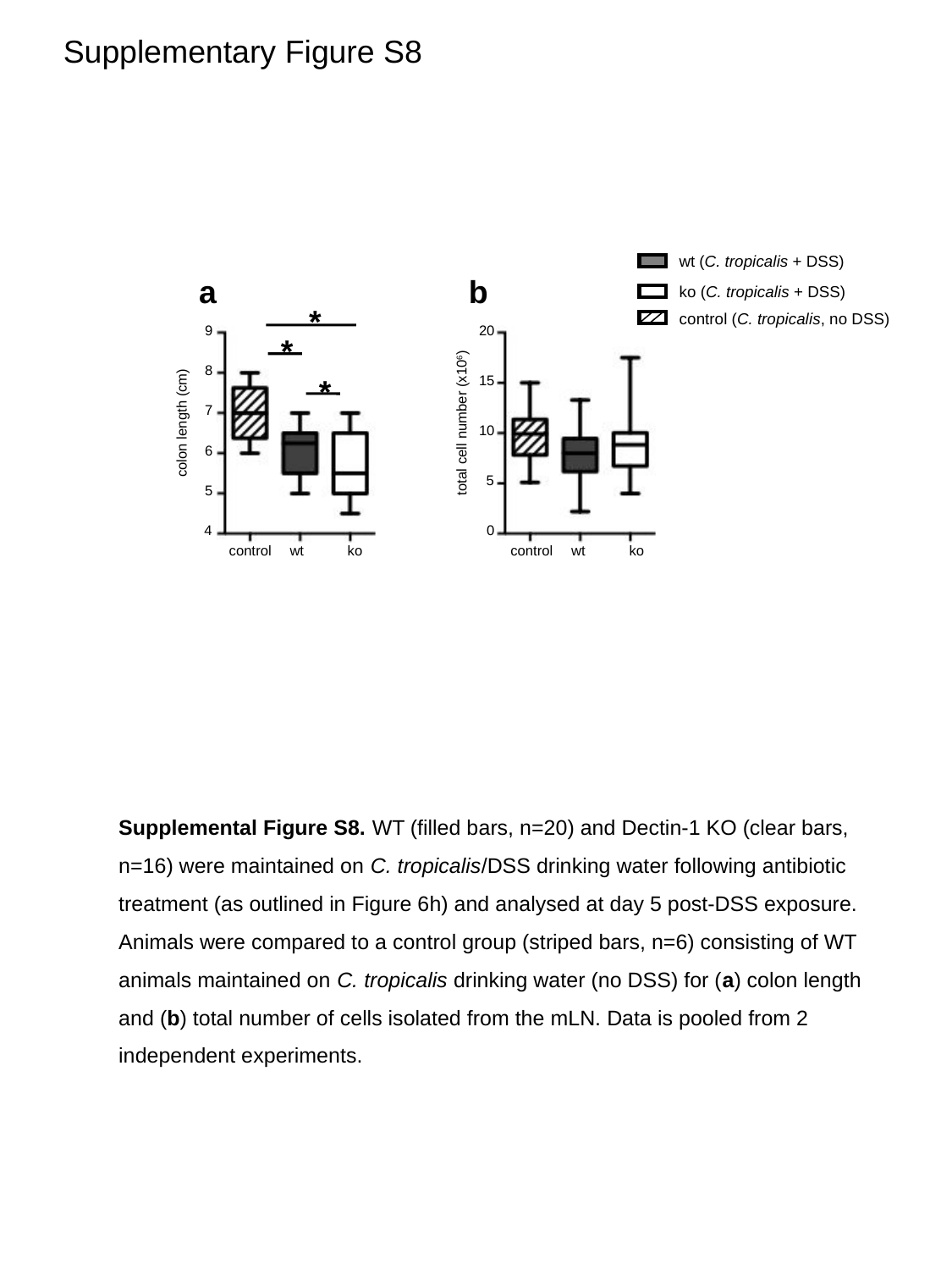

Supplementary Figure S8
wt (C. tropicalis + DSS)
a
b
ko (C. tropicalis + DSS)
*
control (C. tropicalis, no DSS)
9
20
*
8
*
15
7
colon length (cm)
total cell number (x106)
10
6
5
5
4
0
control
wt
ko
control
wt
ko
Supplemental Figure S8. WT (filled bars, n=20) and Dectin-1 KO (clear bars, n=16) were maintained on C. tropicalis/DSS drinking water following antibiotic treatment (as outlined in Figure 6h) and analysed at day 5 post-DSS exposure. Animals were compared to a control group (striped bars, n=6) consisting of WT animals maintained on C. tropicalis drinking water (no DSS) for (a) colon length and (b) total number of cells isolated from the mLN. Data is pooled from 2 independent experiments.

## Slide 9
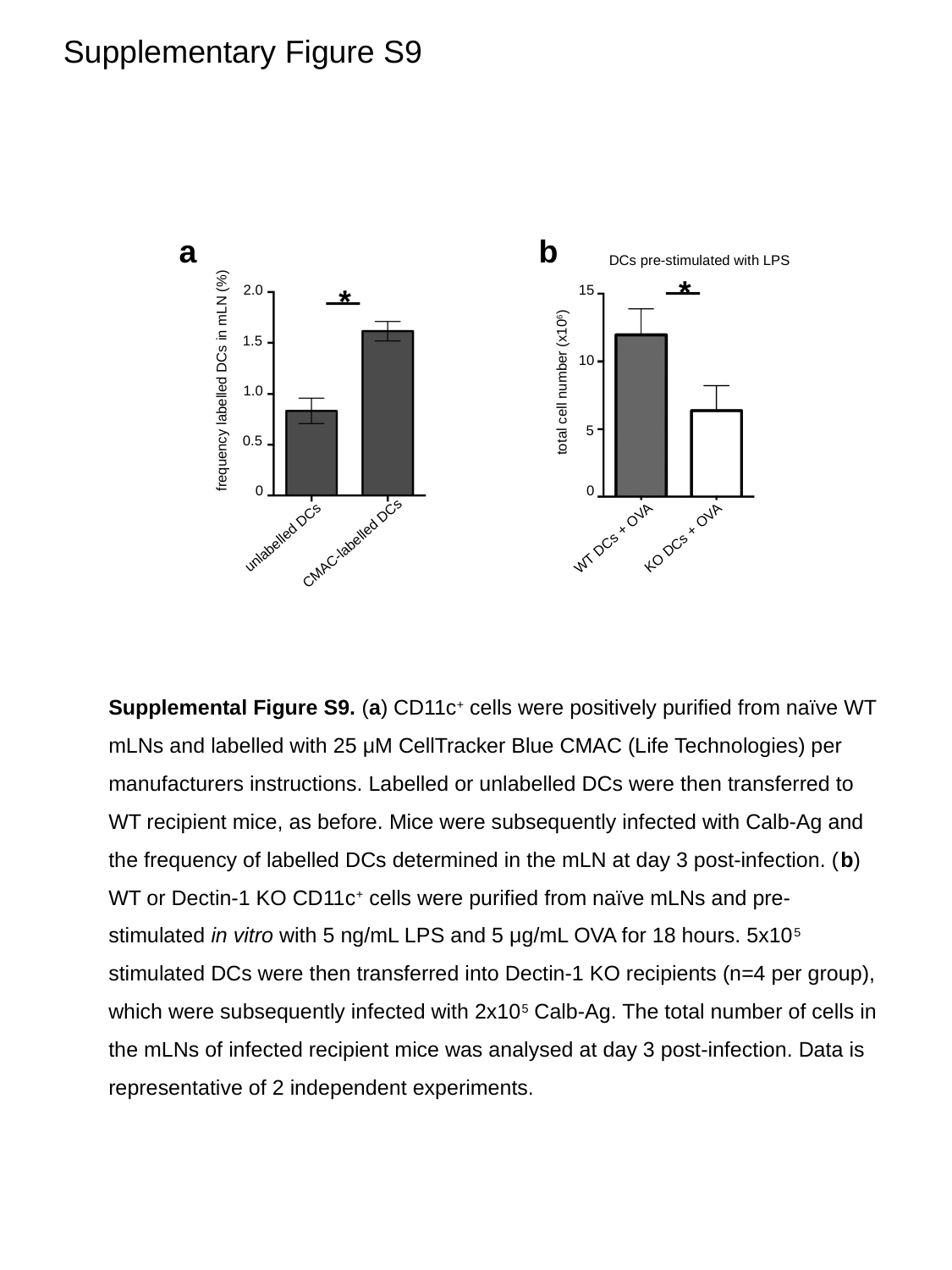

Supplementary Figure S9
a
b
DCs pre-stimulated with LPS
*
2.0
*
15
1.5
10
frequency labelled DCs in mLN (%)
total cell number (x106)
1.0
5
0.5
0
0
unlabelled DCs
KO DCs + OVA
WT DCs + OVA
CMAC-labelled DCs
Supplemental Figure S9. (a) CD11c+ cells were positively purified from naïve WT mLNs and labelled with 25 μM CellTracker Blue CMAC (Life Technologies) per manufacturers instructions. Labelled or unlabelled DCs were then transferred to WT recipient mice, as before. Mice were subsequently infected with Calb-Ag and the frequency of labelled DCs determined in the mLN at day 3 post-infection. (b) WT or Dectin-1 KO CD11c+ cells were purified from naïve mLNs and pre-stimulated in vitro with 5 ng/mL LPS and 5 μg/mL OVA for 18 hours. 5x10^5^ stimulated DCs were then transferred into Dectin-1 KO recipients (n=4 per group), which were subsequently infected with 2x10^5^ Calb-Ag. The total number of cells in the mLNs of infected recipient mice was analysed at day 3 post-infection. Data is representative of 2 independent experiments.
